# Augmentation Therapies as Treatments for Coexisting Somatic Problems in Schizophrenia—A Systematic Review

**DOI:** 10.3390/jcm12124012

**Published:** 2023-06-12

**Authors:** Wiktor Dróżdż, Michał Wiciński, Anna Maria Szota, Monika Szambelan, Izabela Radajewska, Igor Popławski, Paweł Wojciechowski

**Affiliations:** 1 Department of Psychiatry, Ludwig Rydygier Collegium Medicum in Bydgoszcz of Nicolaus Copernicus University in Toruń, Curie Skłodowskiej Street 9, 85-094 Bydgoszcz, Poland; wikdr@cm.umk.pl (W.D.); izabela.radajewska@cm.umk.pl (I.R.); 2 Department of Pharmacology and Therapy, Ludwig Rydygier Collegium Medicum in Bydgoszcz of Nicolaus Copernicus University in Toruń, Curie Skłodowskiej Street 9, 85-094 Bydgoszcz, Poland; michal.wicinski@cm.umk.pl (M.W.); monika.szambelan@cm.umk.pl (M.S.); igorpoplawski19@gmail.com (I.P.); pawel.wojciechowski@cm.umk.pl (P.W.)

**Keywords:** schizophrenia, augmentation therapy, antipsychotics, somatic illness

## Abstract

The aim of this review is to appraise the data from available randomized clinical trials (RCT) regarding the possible combinations of neuroleptic and non-antipsychotic treatment which could enhance antipsychotic therapy efficacy whilst simultaneously addressing somatic symptoms in individuals with schizophrenia. A systematic search of the PubMed database up to February 2022 was conducted. Inclusion criteria: randomized controlled trials using augmentation therapy in chronic schizophrenia in adults, written in English, and only studies with psychometric assessments of schizophrenia were incorporated. Exclusion criteria: non-clinical, first episode of schizophrenia, patients on medication other than antipsychotics augmented, and not adjunctive therapy. Overall, 37 studies of 1931 patients with schizophrenia who received a combination of antipsychotic medication with other drugs were selected. A statistically significant reduction of negative and positive symptoms of schizophrenia, measured with the PANSS scale, when using a combination of antipsychotic treatment along with aspirin, simvastatin, N-acetylcysteine, or pioglitazone was found. A combination of antipsychotic medication with aspirin, simvastatin, N-acetylcysteine, or pioglitazone seems to be effective in the reduction of symptoms of schizophrenia in adults, but long-term studies are required to confirm this effect.

## 1. Introduction

Schizophrenia is one of the most serious mental health disorders which usually manifests with the disorganization of many mental functions associated with positive, negative, cognitive, and affective symptoms, and can therefore cause serious difficulties for a patient and their daily activities. Lifetime prevalence in the general population is estimated at c.a. 1% [1,2]. The prevailing unsatisfactory effectiveness of schizophrenia therapeutic methods is reflected in the results of various studies, which indicate that 20–50% of patients meet the criteria for drug resistance [1]. This observation is calculated from the meta-analyses of the effect size for psychopharmacological interventions in schizophrenia, estimated at 0.38 [3]. Insufficient effectiveness of therapeutic methods in schizophrenia generates significant costs, not only economic but also social [2,4]. Hence, augmentation strategies for antipsychotic therapies have substantial importance. A thorough analysis by Corell et al. [5] concluded that the quality of studies in this field should be regarded as unsatisfactory, however, the evidence indicates a fairly wide range of medications that could be possibly used as antipsychotic enhancers.

Various somatic conditions coexist in many patients with schizophrenia, sometimes the side effects of antipsychotic therapy, especially with the use of second-generation agents. Such conditions include hypertension, metabolic syndrome, diabetes, hormonal disorders, chronic obstructive pulmonary disease (COPD), and other inflammatory diseases [6,7,8]. Individuals suffering from schizophrenia experience a substantially shorter lifespan compared to the general population, estimated between 15–20 years less, which derives from both lifestyle factors and the side effects of neuroleptic therapies. Among many other reasons, suicide as well as smoking have a substantial impact and should be considered clinically important [9,10,11].

Drug repositioning or repurposing is a strategy aimed at identifying new indications for already registered medications. This procedure nowadays relies on a deep exploration of bioinformatics and pharmacogenomics data and is backed by machine learning algorithms [12,13]. A retrospective clinical data analysis of pharmacologic data is another method, which has been also employed [14]. Drug repositioning, repurposing, or combining has become regarded as a potentially valuable approach to dealing with the numerous obstacles in new drug discovery and, concurrently, with the matter of drug resistance in neuropsychiatric conditions. The high and growing prevalence of these conditions, as well as their financial and social consequences, constitute a big challenge to public health [12,15]. Not surprisingly, this method has attracted increasing attention from scientists and has generated noteworthy results for many years. A well-known example is amantadine, originally invented as a drug for type A influenza, then repurposed as a medication for Parkinson’s disease [16,17].

This review aims to assess the data from current clinical trials and cohort studies on the possible combinations of antipsychotics with medications used for the treatment of somatic diseases in schizophrenic patients, in order to achieve two key targets: (i) the effective treatment of frequent coexisting somatic problems and (ii) the augmentation of the antipsychotic effect. Considerate collation of the data regarding neuroleptic treatment with other drugs may potentially affect the modifiable factors that influence the life expectancy of patients with schizophrenia, i.e., metabolic syndrome. Such a strategy may have beneficial clinical outcomes, including benefits from a public health perspective.

## 2. Materials and Method

### 2.1. Protocol and Registration

The current review followed the preferred reporting item for systematic review and meta-analysis criteria (PRISMA 2020) [18]. The protocol was registered by the International Prospective Register of systematic reviews (PROSPERO), registration number: CRD 42023391014.

### 2.2. Search Process

Although, according to Bramer et al. [19], medical searches should be performed on more databases, our study employed PubMed exclusively because, consistent with Halladay et al., [20] findings, in the field of RCT studies exploring data sources beyond this database “has a modest impact on the results of systematic reviews of therapeutic interventions”.

The PubMed database was searched until 28 February 2022, using the following key terms: ‘schizophrenia’ and ‘augmentation therapy’; ‘schizophrenia’ and (aspirin OR simvastatin OR statins OR N-acetylcysteine OR pioglitazone OR estrogens OR raloxifene OR memantine OR ondansetron OR telmisartan). Additional articles were searched to detect similar reports and those with titles containing our search terms. Articles were selected using a two-stage process: title and abstract screening and full-text assessment.

### 2.3. Data Extraction

Titles and abstracts were screened (W.D.; M.W.) and full-text articles were further assessed for eligibility in an independent manner (A.S.; P.W.). Data extraction of full-text articles was performed by three independent coders (W.D.; A.S.; P.W.). The selection of the articles was conducted on the basis of the inclusion and exclusion criteria.

### 2.4. Study Inclusion and Exclusion Criteria

**Figure 1:**
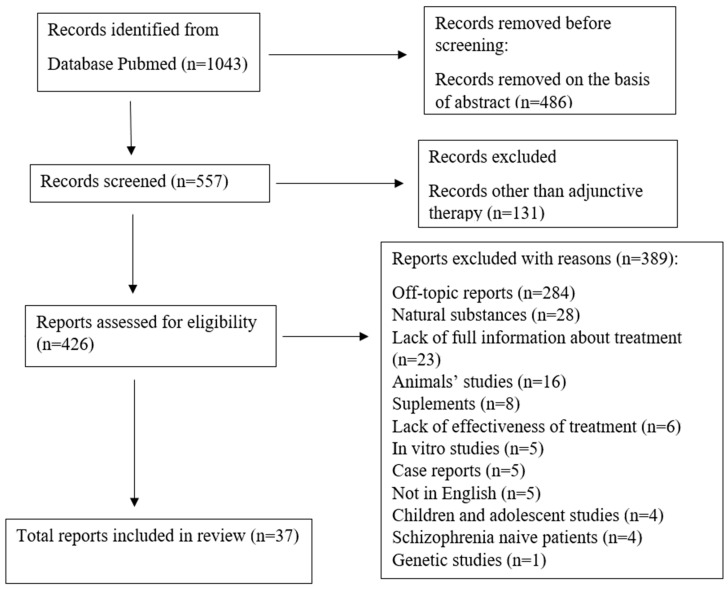
PRISMA 2020 flow chart for systematic reviews. Identification of new studies via databases and registers.

The following inclusion criteria were applied: full-text articles available in English; studies only in humans; only randomized controlled trials; only trials considering augmentation therapy in schizophrenia; not supplementary therapy; and studies providing detailed drug information (name and dose of the drug, route of administration, length of treatment, effectiveness of treatment). Articles that describe research conducted on animals, in vitro, and among children and adolescents were excluded. Further exclusion criteria were articles about genetic research; case reports; patients with a first episode of schizophrenia, drug-naive schizophrenia as a somatic burden in younger patients; current use of other medication other than antipsychotic therapy, not as the augmentation therapy; use of supplements, natural substances, and vitamins; and describing a lack of effectiveness in schizophrenia. Additionally, off-topic articles were excluded by title and abstract. A diagram of the identification of the randomized controlled trials included in the review is presented in Figure 1.

### 2.5. Risk of Bias Assessment

The risk of bias (RoB) of the included studies was assessed on the basis of study quality (quantitatively) by answering the following Yes/No-criteria:

1. Control-comparison: Was there a control group or comparator to which adjunctive therapy was compared?
2. Ordering/assignment control: Was it assured that the interventions or intervention groups being compared were randomized or counterbalanced?
3. Pre/post-comparison: Was there a pre-measurement to which the post-measurement was compared?
4. Follow-up: Was there a follow-up measurement after weeks?
5. Registration: Was the study enrolled in an official study register?
6. Experimenter-blinding: Were the study conductors blinded with respect to the interventions or intervention groups being compared?
7. Analyst-blinding: Were the study evaluators blinded with respect to the interventions or intervention groups being compared?
8. Participant-blinding: Were the participants naive to whether they received an actual intervention or a control intervention?

Based on the Yes/No-criteria, a risk of bias score was evaluated by calculating the percentage of unfulfilled criteria. A 0% RoB indicates a high study quality and a 100% RoB indicates a low study quality. This method has been recently elaborated and applied by Wiebe et al. [21]. In the case of meta-analyses, each clinical trial included was evaluated separately. The results of the quantitative assessment of each randomized clinical trial are presented in Table S1 (attached as a Supplementary File).

## 3. Results

The search of the PubMed database yielded 1043 total records. After removing 486 records on the basis of abstract content and an additional 131 records describing other therapy than adjunctive, 426 records were left for eligibility. Of these, 37 reports met the full inclusion criteria and were included in this review, as shown in Table 1. In the case of the meta-analysis summary, a number of RCTs for particular drugs were found.

The quality of studies considered *n* = 37 (risk of bias), evaluated on the basis of Yes/No questions, revealed that four out of thirty-seven studies (10.81%) had the highest standards (no risk of bias). Additionally, 23 out of 37 studies (62.16%) had high standards and only the lack of follow-up was a disadvantage in these studies (low risk of bias). Further lowering of quality of included studies resulted from both the lack of follow-up and the lack of registration in, seven out of thirty-seven studies (18.91%). Three out of thirty-seven studies (8.10%) were evaluated as moderate quality (moderate risk of bias). In these reports, apart from the lack of follow-up, issues combined with blinding (the lack of experimenter, analyst, and participant blinding) were found. The quality of the studies varied; however, the aim of this review is to assess the data from current clinical trials on the possible combinations of antipsychotics with medications used to treat somatic diseases in schizophrenic patients in order to point to the effective treatment of frequently coexisting somatic problems and to augment the antipsychotic effect of neuroleptics. Therefore, all study designs and all levels of quality were included.

## 4. Discussion

The results of many clinical studies indicate that combining antipsychotics with other medications to decrease the intensity of psychopathological symptoms of schizophrenia and simultaneously achieve effective treatment of comorbid somatic burdens may be considered a valuable therapeutic option. Most observations concern the possible effects of such augmentation on the severity of negative and general symptoms of schizophrenia (Table 1).

### 4.1. Non-Steroidal Anti-Inflammatory Drugs (NSAIDs)

Aspirin is a non-steroidal, anti-inflammatory drug that modifies the activity of cyclooxygenase 2 (COX-2). It is also an irreversible cyclooxygenase 1 (COX-1) inhibitor. This leads to a reduction in prostaglandins and thromboxane production, which are involved in inflammatory processes. A neuroinflammatory response translates into the activation of microglia, which increases the production of cytokines, especially interleukin 6 (IL-6), the increase of which is evident in patients during the first episode or in the acute relapse of schizophrenia [62]. The action of taking aspirin also leads to a reduction in the activation of the hypothalamus-pituitary-adrenal stress axis (HPA), which is responsible for an increased level of pro-inflammatory cytokines and, thus, may exacerbate the symptoms of schizophrenia [63].

The blood-brain barrier is poorly permeable for aspirin, but with inflammation, its ability to penetrate the central nervous system (CNS) is increased. Aspirin concentration in the CNS is always lower than in peripheral blood circulation [64]. Therefore, its use in higher doses, necessary to achieve a therapeutic concentration in the CNS, may exacerbate adverse effects connected with coagulation and the digestive system. Despite this, adjuvant aspirin therapy, in addition to the main antipsychotic treatment, may turn out to be an inexpensive and effective clinical intervention.

A meta-analysis of randomized, placebo-controlled trials involving 70 patients has shown that the addition of aspirin to antipsychotic therapy for 3 months, at a dose of 1000 mg per day, has a positive effect on the severity of the psychopathological symptoms of schizophrenia. The mean weighted effect size (ES) of the intervention = 0.30, which is statistically significant. The analysis has revealed a mean difference of a score decrease of 4.86 points on the total PANSS scale and 1.86 points on the positive symptoms PANSS subscale in patients using aspirin compared to the placebo group. A greater reduction in symptom severity in the group receiving aspirin was also shown on other PANSS subscales, but the differences were not statistically significant. Interestingly, the most pronounced decrease in the severity of schizophrenia symptoms was observed in patients with the strongest deviations in the assessment of immunological parameters. There were no significant differences in the efficiency of cognitive functions between the studied groups [35]. It should be noted that there is a lack of serious side effects using aspirin therapy. Nevertheless, the evaluation was performed on a small group of patients, and the short observation period does not allow us to estimate the risk of the long-term side effects that may be related to the long-term use of the drug, including the risk of gastrointestinal ulceration and hemorrhagic symptoms.

The effectiveness of this intervention can be explained in the context of the inflammatory hypothesis of schizophrenia by the inhibition of the COX-2 enzyme by aspirin, which decreases the concentration of kynurenic acid and its metabolites in the CNS. These factors are associated with the enhancement of glutamatergic activity, microglia activation, and the formation of pro-inflammatory cytokines, chemokines, and proteases [65]. Aspirin is a prodrug, metabolized into its active form by the following enzymes: at low doses, 90% of the drug is transformed by N-acetyltransferase (NAT2) and glucuronosyltransferase (UGT1A6), while at high doses, by cytochrome P450 2C9 (CYP450 2C9). As a result, the risk of interactions with antipsychotics, which are mainly metabolized by CYP450 2D6, is low. For these reasons, no negative effects of aspirin on the metabolism or interaction with antipsychotic drugs have been reported. Nevertheless, the polymorphism of the genes responsible for the production of aspirin to metabolize enzymes accounts for some interindividual variability in its pharmacokinetics and pharmacodynamics.

Psychotic disorders have been shown to be related to an increased risk of short-term mortality following acute coronary syndrome (ACS) [66]. Taking into account the extensive application of aspirin in the primary and secondary prevention of coronary artery disease, it seems interesting whether aspirin added to antipsychotic therapy could reduce this risk and, thus, extend the life span of patients with schizophrenia. There is no evidence in this regard in the available literature, therefore, it is reasonable to expect that aspirin, as a cheap and potentially effective adjunctive therapy in the treatment of schizophrenia, requires further research.

### 4.2. Other Anti-Inflammatory Drugs

The main drugs used in the secondary prevention of cardiovascular diseases (CVD) are statins. It has been noted that their use in patients with schizophrenia may reduce the severity of negative symptoms, but the differences are not statistically significant [23,29,30]. The anti-inflammatory effect of statins works by reducing the production of inflammatory cytokines, such as interleukin 1-β (IL1-β), interleukin 6 (IL-6), tumor necrosis factor α (TNF-α), and C-reactive protein (CRP) [67,68,69], and it is hypothesized that the lipophilicity of statins improves the effects of treatment with antipsychotic drugs. Simvastatin and atorvastatin have shown a greater ability to cross the blood-brain barrier compared to rosuvastatin and pravastatin.

Statins are the main drugs for the secondary prevention of cardiovascular diseases (CVD). It has been noted that their use in patients with schizophrenia may reduce the severity of negative symptoms, but the differences are not statistically significant [23,29,30]. A study by Ghanizadeh et al. [26], evaluating the effects of adding lovastatin, at an initial dose of 10 mg and then increasing to 20 mg, to risperidone, did not show a statistically significant change as the PANSS total score decreased in the study and control groups, and the difference between them was not significant (*p* = 0.3), [26]. The addition of simvastatin (40 mg/d) to risperidone (6 mg/d) over 8 weeks in the Tajik-Esmaeelii et al. study [27] resulted in a reduction of negative symptoms at the end of the study (*p* = 0.003), while the decrease of positive symptoms did not differ significantly in both subgroups, and there were no effects on depressive symptoms and extrapyramidal symptoms [27]. Reduction of the negative symptoms and a lack of influence on positive symptoms was also observed when adjunctive therapy of simvastatin (40 mg/d) with risperidone (6 mg/d) was extended to 6 months of treatment [30]. Chaudhry et al.’s study [28] evaluated the addition of simvastatin at an initial dose of 20 mg, which increased to 40 mg after 12 weeks. The combination of standard therapy and simvastatin was well tolerated. However, there were no significant differences between the groups. The only effect of the therapy was the reduction of the overall and positive scores, but no differences were found in the reduction of negative symptoms [28]. Similar results were observed when pravastatin (40 mg/d) was added to antipsychotic drugs for 12 weeks. Further treatment after 12 weeks showed that pravastatin failed to retain its effects on schizophrenia symptoms [25]. In the Sayyah et al. study [29], evaluating the effect of atorvastatin (20 mg/day) as an addition to risperidone (6 mg/day), no beneficial effect on the reduction of symptoms in patients with schizophrenia was found [29].

The above findings indicate that the effectiveness of statins as an adjunctive therapy to antipsychotic drugs depends mostly on what statin (simvastatin, parvastatin, atorvastatin) was used. The most effective combination in the reduction of schizophrenia symptoms was the addition of statins to risperidone and conducting 8 weeks of treatment with these drugs. Furthermore, statins had a significant influence on the negative symptoms, whereas their impact on other schizophrenia symptoms (positive and cognitive symptoms) was differentiated. The different number of patients in these groups does not seem to affect the results obtained.

In a meta-analysis performed by Shen et al. [24] six randomized clinical trials (RCTs) were analyzed, involving a total of 339 patients, divided into subgroups depending on the type of statin and antipsychotic drug used. Patients receiving statins showed a significant reduction in scores on the positive as well as the negative symptoms PANSS subscale, wherein the best effects in reducing negative symptom severity were noted in patients taking simvastatin. It is suspected that the pharmacokinetic interaction between simvastatin, P-glycoprotein, and neuroleptics may be of importance. P-glycoprotein is located within the blood-brain barrier and influences the concentration of neuroleptics in the cerebrospinal fluid. In vitro studies have shown that risperidone and quetiapine have a high affinity for P-glycoprotein, while indirect affinity is characteristic of chlorpromazine and olanzapine, and the lowest for haloperidol and clozapine. Statins are substrates of this protein and have the ability to block it, increasing the penetration of the mentioned drugs into the cerebrospinal fluid, hence, this may lead to a remarkable reduction in the severity of negative symptoms in patients with schizophrenia when taking simvastatin with risperidone [27]. In turn, the combination of risperidone with atorvastatin does not result in significant changes in the severity of schizophrenic symptoms [29].

### 4.3. Antioxidants: N-acetylcysteine (NAC)

N-acetylcysteine (NAC) is an inflammation-modulating drug that can easily penetrate the blood-brain barrier. Its action is based on inhibiting the tumor necrosis factor (TNF-α), interleukin 1β (IL-1β), and interleukin 6 (IL-6) biosynthesis [70] and by increasing the concentration of glutathione. The pleiotropic effect of NAC indicates the possibility of affecting patients with schizophrenia through several mechanisms. Three studies on the use of NAC as an adjunctive therapy in schizophrenic patients showed a significant improvement in scores on the PANSS scale.

In the Iranian study, patients with schizophrenia received NAC 2 g/day or placebo in addition to risperidone therapy (up to 6 mg/day) for 8 weeks. In total, 140 people were enrolled in the study, but only 84 participants completed it. Patients receiving NAC showed a remarkably greater reduction in the total PANSS score (*p* = 0.006) as well as a reduction in the severity of negative symptoms (*p* < 0.001) compared to the placebo group. However, no differences in scores were found on the subscales of positive and general symptoms of PANSS [32]. Another study estimated the efficacy of adding 1200 mg NAC to treatment with first-generation antipsychotics in 82 patients with chronic schizophrenia over 12 weeks. Patients receiving NAC achieved a significant reduction in scores on the PANSS positive (*p* = 0.02) and negative (*p* = 0.001) symptoms subscales. This group also showed improvements in cognitive functions such as memory, attention, and information processing speed [33].

Other studies confirmed the efficacy of the NAC added to neuroleptic therapy. Berk and colleagues (2008) [31] found that a combination of 2 g/day NAC with risperidone for 24 weeks decreased the intensity of positive and negative symptoms on the PANSS (d = 0.52), as well as general symptoms (d = 0.46) and overall PANSS scores (d = 0.57) as compared with the placebo. Interestingly, akathisia intensity also significantly decreased in the intervention group. Moreover, this study showed the superiority of the 24-week over the 8-week combined therapy [31].

The presented studies show the effectiveness of NAC as an addition to standard therapy. Moreover, longer exposure to combination therapy results in better outcomes. Additional benefits of this intervention are the lack of adverse effects of NAC both at the beginning and at the end of the study, as well as the mitigation of side effects of standard therapy. It is worth noting that the intervention appeared effective both in outpatients and hospitalized patients. Factors such as sex, dosage, and duration of the disease had no significant effect.

An additional benefit of N-acetylcysteine use in patients with schizophrenia may be in helping to cope with nicotine addiction. It is estimated in the meta-analyses from the UK that about 45% of people diagnosed with schizophrenia are smokers [71]. Smoking cigarettes contributes to an increased cardiovascular risk and premature mortality. The induction of hepatic cytochromes in smokers leads to a reduction in the concentration of most antipsychotics [72]. This is an important factor because higher doses of schizophrenia drugs are then required, which in turn increases the risk of side effects. In the case of second-generation antipsychotics, metabolic syndrome, in combination with nicotine, results in a significantly increased risk of cardiovascular diseases. Hence, smoking is an important predictive factor for these patients. In this context, it is worth mentioning a randomized, blind study in which smokers (without psychiatric burdens) not seeking treatment received 2400 mg of NAC or a placebo. Individuals treated with NAC maintained abstinence and declared less desire to smoke and a higher level of positive affect (*p* < 0.01) compared to the placebo [73]. Similar effects were observed in a 12-week study in which NAC was combined with the first-line drug in psychiatrically unburdened subjects addicted to tobacco. Moreover, NAC therapy was not associated with serious side effects, suggesting that the drug is safe [74]. Therefore, a reduction in the severity of psychopathological symptoms and help in coping with nicotine constitutes a double potential benefit in people suffering from schizophrenia.

### 4.4. Peroxisome Proliferator-Activated Receptor Gamma (PPARγ) Agonists

Pioglitazone, one of the peroxisome proliferator-activated receptor gamma (PPARγ) agonists, is an antidiabetic drug with anti-inflammatory and antioxidant properties. In a placebo-controlled study in a group of 40 patients treated with risperidone at a dose of 6 mg/day, pioglitazone, at a dose of 30 mg/day, was used and assessed to determine its ability to decrease the severity of schizophrenia symptoms. After 8 weeks, the mean score decrease on the PANSS negative subscale was 5.55 (±2.9) in the pioglitazone group and 2.3 (±2.3) in the placebo group, and the difference was statistically significant (*p* < 0.001). There was no significant difference between these two groups in the score change for the positive symptoms subscale at the endpoint [34]. However, there are numerous reports of the possible side effects of pioglitazone pharmacotherapy, such as cardiac complications, water retention, weight gain, bladder cancer, and reduction in bone mineral density leading to fractures, which resulted in a clear downward trend in the use of this drug [75]. Therefore, it seems reasonable to state that patients with schizophrenia, using antipsychotic drugs that also show cardiotoxic effects, should avoid the inclusion of further drugs with a similar profile of side effects.

### 4.5. Estrogens

Taking into account the fact that the pathogenesis of schizophrenia is associated with disturbances in dopaminergic and serotonergic transmissions, dysregulation in the immune system, as well as neurodegenerative processes that progress with the duration of the disease, research has been undertaken on the use of estrogen drugs which could be added to antipsychotic therapy. Estrogens demonstrate neuroprotective and immunomodulatory effects [76], reduce oxidative stress, control energy balance and glucose levels, and affect dopaminergic and serotonergic transmissions [36]. The efficacy of estrogens added to antipsychotic treatment in patients with chronic schizophrenia has been assessed in 11 clinical trials [36,37,38,39,40,41,42,43,44,45,46]. Nine were conducted in women (*n* = 368) and two were conducted in men (*n* = 49), [38,43]. In recent years, the efficacy of raloxifene (a selective estrogen receptor modulator) combined with antipsychotic drugs in male and female patients suffering from chronic schizophrenia has also been tested [38,43,44,45,77].

The addition of transdermal estradiol (100 mg/day) [36] or estradiol (100 µg/day) [37] to an atypical antipsychotic produced greater improvement in schizophrenia symptoms measured with the PANSS scales and subscales (*p* < 0.005) in females as compared to women treated only with antipsychotic drugs for 28 days. Additionally, the improvement of general functioning evaluated with the General Psychopathology scale (*p* < 0.005) was observed. A similar result was obtained with a combination of estradiol (0.05 mg/day) and haloperidol (15 mg/day) in women with chronic schizophrenia during 8 weeks of treatment (*p* < 0.0001) [39]. Interestingly, a combination of estradiol with antipsychotics for 2 weeks decreased schizophrenia symptoms in men compared to the placebo (*p* < 0.0001) [38]. Treatment of schizophrenia with conjugated estrogens (0.625 mg/day) added to haloperidol (5 mg/day) [40] or to atypical antipsychotic drugs [41] for 28 days decreased the severity of positive schizophrenia symptoms on the BPRS (*p* < 0.065) or the PANSS (*p* < 0.001) scales and improved general functioning of patients, but it did not cause a clinical improvement of negative symptoms (NSRS scale). The above-described findings on the effectiveness of estrogens added to first- and second-generation antipsychotics are coherent, however, it is worth noting that the duration of treatment was short (14 days to 8 weeks).

The effectiveness of another neuroprotective drug, raloxifene (a selective estrogen receptor modulator) as adjunctive therapy to antipsychotics in patients with schizophrenia or schizoaffective disorder was also examined in clinical trials [42,43,44,45,46]. A combination of raloxifene (120 mg/day for 8 weeks) with risperidone (6 mg/day) improved the general functioning of females with schizophrenia [MD: (95 %CI) = −7.9 (−12.8 to −3.1), t(40) = −3.327, *p* = 0.002] in comparison to the control group treated with risperidone only [43]. In regard to the positive symptoms, Kianimehr et al. [42] found that adjunctive therapy reduced severity [F (1, 48) = 3.78, *p* = 0.02], however, Khodaie-Ardakani et al. [43] did not observe a significant difference [MD: (95% CI) = −1.6 (−6.5 to 3.4), t (40) = −0.641, *p* = 0.525].

A significant reduction in the severity of negative symptoms was found in men, (MD: (95% CI) = −6.3 (−9.4 to −3.3), t(40) = −4.183, *p* < 0.001] [43]. However, this effect was not observed in women [F (1, 48) = 1.43, *p* = 0.23) [42]. Another study with raloxifene (120 mg/day) combined for 12 weeks with antipsychotics in patients with schizophrenia or schizoaffective disorder found an improvement in functioning on the General Psychopathology Scale [TE −3.72 95% CI (−6.83 to −0.61); *p* < 0.02], but a marginal effect was seen on the positive and negative symptoms of schizophrenia [β = −1.92; 95% CI, −3.83 to 0.00; *p* = 0.05] [44]. An extended treatment of up to 24 weeks with a lower dose of raloxifene (60 mg/day) added to antipsychotics ameliorated the positive and negative symptoms of schizophrenia (*p* = 0.027) [45]. However, Weiser et al. [46] reported that raloxifene, at 120 mg/day for 16 weeks, added to antipsychotics was not effective in reducing the symptoms of schizophrenia in severely ill, decompensated postmenopausal women [46].

The above findings indicate that the efficacy of the addition of raloxifene in reducing the severity of symptoms of schizophrenia depends primarily on the duration of observation and, to a lesser degree, on its dose. Factors such as the age of patients (18–55), disease duration, type of antipsychotic drug (first or second generation), and the specific phase of the menstrual cycle in women did not have a significant influence on the effectiveness of the intervention. In addition, the lack of effectiveness of raloxifene might result from the enrolment of severely decompensated female patients with chronic schizophrenia of a postmenopausal age.

Findings of these clinical trials indicate that the addition of estrogens to neuroleptic treatment in schizophrenic patients of both sexes may have a beneficial effect on the reduction of symptoms. However, this effect was observed only in short-term and medium-term treatment, and only in small groups of patients in whom the levels of sex hormones were not measured. This is especially important in women due to the changing hormone balance in the pre- and post-menopausal periods. Furthermore, the long-term use of antipsychotic drugs often causes hormonal side effects such as hyperprolactinemia, menstrual disorders, ovulation suppression, and gynecomastia [77,78]. Antipsychotic-induced hyperprolactinemia leads to hypogonadism, infertility, decreased libido, irritability, bone calcification disorders, and depressive states in both sexes [79,80]. The addition of estrogens in the form of tablets, skin patches, as well as a component of hormone replacement therapy (HRT) in women, may further increase prolactin levels and worsen the side effects of antipsychotics, which were described above [78]. Moreover, previous studies involving women showed that the use of HRT, at least 10 years after menopause or after the age of 60, significantly increased the risk of heart attack, cancer development, and thromboembolism [81,82]. Hence, introducing this therapy to female patients suffering from schizophrenia without the constant monitoring of sex hormone levels may turn out to be risky. The use of HRT in female smokers is particularly dangerous as it may increase blood clotting and, thus, the risk of thromboembolic complications, including stroke [83,84]. Given the above data, the short-term improvement of the mental state of patients (reduction of the severity of positive and/or negative symptoms) after adding estrogens to neuroleptic treatment cannot be considered more important than the potential serious side effects of such therapy. For this reason, the use of estrogens in patients with schizophrenia should not be recommended.

### 4.6. N-methyl-D-aspartate Receptor (NMDA) Antagonists

Multiple preclinical and clinical studies confirm a link between glutamatergic neurotransmission and schizophrenia, giving a foundation for the use of memantine, as mainly an adjunct to antipsychotic drugs. The addition of memantine (20 mg/day) for 12 weeks [48] to clozapine in patients with treatment-resistant schizophrenia resulted in significant improvements (*p* < 0.01) in the total score of the Brief Psychiatric Rating Scale (BPRS), its positive (ES = −1.38) and negative symptom subscales (ES = −3.33), the Clinical Global Impression (CGI) score (ES = 1.56,) and the Mini-Mental State Examination (MMSE) score compared with the placebo. Similar therapeutic effects were obtained with memantine at a dose of 10 mg/day and increasing this after 1 week to 20 mg/day taken for 11 weeks as an add-on therapy to ongoing clozapine treatment [54]. There were improvements in verbal recognition memory and paired learning task scores on the CANTAB (ES = 0.30) and a negative PANSS subscale score (ES= 0.29). In a study of 64 patients taking atypical antipsychotics [52], memantine was started at a dose of 5 mg/day and increased by 5 mg at weekly intervals, eventually increasing to 20 mg/day over 4 weeks. The Global Assessment of Functioning (GAF) and Quality of Life Scale (QLS) scores increased in both the placebo and study groups but were higher in the group with memantine (*p* < 0.001 and *p* < 0.001, respectively). The efficacy of memantine, in combination with olanzapine, has also been tested [53]. Patients received memantine (week 1: 10 mg/day; weeks 2–6: 20 mg/day) plus olanzapine (15–20 mg/day) or olanzapine in combination with the placebo. Memantine significantly improved the PANSS scores on the positive and negative symptoms subscales (*p* < 0.001). It was also shown that women showed a better response than men, especially in the positive PANSS score. Treatment of the primary negative symptoms in patients with schizophrenia stabilized on risperidone and memantine (20 mg/day) for 8 weeks, showing that patients in the intervention group had a significantly greater improvement in the negative subscale than the placebo group (*p* < 0.001) [51]. The same effect was observed for the total score (*p* < 0.001) and the overall psychopathology subscale score (*p* = 0.002). There was no significant difference in the reduction of the positive symptoms score between the placebo and memantine groups (*p* = 0.757). The same was true for the Hamilton Depression Rating Scale and Extrapyramidal Symptom Rating Scale scores and the incidence of adverse effects in the two groups. The study showed that memantine is a tolerable and effective adjunctive treatment for the primary negative symptoms of schizophrenia [51]. A study by Mazinani et al. [55], which evaluated the effect of memantine (20 mg/day) as an adjunct to risperidone in men with schizophrenia, reached similar conclusions [55]. Furthermore, in this trial, a significant improvement in the negative symptoms was observed at week 12 of treatment. Cognitive functions also improved. The addition of memantine to risperidone did not result in differences between positive and general psychopathological symptoms compared to the placebo at baseline and after treatment.

Based on these studies, it appears that memantine may be an effective adjunctive treatment to improve negative and cognitive symptoms in patients with schizophrenia. The above findings are consistent in terms of the dose of memantine, the beneficial effects on cognitive functions, and also the lack of serious side effects in patients with schizophrenia.

Interestingly, in other clinical trials using the addition of memantine (20 mg/day) to atypical antipsychotics for 8 weeks [49] and 12 weeks [50], patients with residual or chronic schizophrenia, respectively, did not show efficacy. At the endpoint, the PANSS total scores did not differ between the memantine and placebo groups (*p* = 0.570, LOCF) [49] and improvements on the PANSS negative subscale were insignificant [50]. Patients with residual schizophrenia had a higher incidence of severe side effects than placebo [49], while in patients with chronic schizophrenia, memantine was well tolerated and did not exacerbate positive symptoms [50]. Possibly, the type of schizophrenia may affect both the efficacy and tolerance of memantine. Furthermore, large differences between the number of participants (e.g., 10 and 70) are apparent in these studies. Factors such as treatment duration and memantine doses were similar in all studies, and therefore, probably did not affect the results.

The results of the meta-analysis conducted by Kishi (2017) [47] indicate that adding memantine to antipsychotic therapy may be beneficial, especially when concerning the negative symptoms of schizophrenia. Animal, genetic, and postmortem studies suggest that glutamatergic receptor dysfunction is associated with the development of positive and negative symptoms of schizophrenia as well as cognitive impairment [85,86]. In eight clinical trials (448 patients, including 239 patients taking memantine (20 mg/day) and 209 receiving a placebo for between 6–26 weeks), it was found that adding memantine to antipsychotic treatment (clozapine, olanzapine, risperidone, aripiprazole, ziprasidone, and first-generation antipsychotics) is well tolerated in both groups of patients. In addition, combination therapy of neuroleptics with memantine was effective in relieving negative symptoms, reflected on the PANSS’s negative subscale (*p* = 0.002). However, there was no reduction in the severity of the PANSS’s general and positive symptoms. An improvement also occurred in the performance of the Mini-Mental State Examination (MMSE test), (*p* < 0.0001). Much smaller differences regarding the influence of memantine on negative and positive symptoms were observed in patients taking memantine and risperidone [51,55]. This means that the observed effect depends on the type of antipsychotic drug [87]. From a clinical point of view, it is difficult to justify such a model of pharmacotherapy based on an average study length of 10 weeks. Therefore, the observed effect may be short-term and clinically irrelevant. Moreover, it should be noted that the majority of patients were young men, and the size of the groups was unsatisfactory. It appears that the addition of memantine to antipsychotic therapy may turn out promising when the aim is to reduce negative symptoms in young adults with schizophrenia. Such a statement, however, should be treated with caution.

### 4.7. Serotonergic Drugs

A therapeutic effect using second-generation antipsychotic drugs may be partially related to their interaction with the serotoninergic 5-HT3 receptor. Case reports [88,89,90] and RCT studies [90] from many years ago already show that ondansetron may be effective in the treatment of psychotic symptoms. Ondansetron is used to treat nausea and vomiting induced by other drugs, for example, chemotherapy.

The administration of ondansetron (8 mg/day) in combination with atypical antipsychotics in patients aged 18–65 years with schizophrenia was assessed using the Positive and Negative Syndrome Scale (PANSS) [28,57,58], the Clinical Global Impression Scale (CGI-S) [28], the Global Assessment of Functioning (GAF) [28] and the Abnormal Involuntary Movement Scale (AIMS) [28]. Patients who received ondansetron had significantly greater improvements in negative symptoms, general psychopathological symptoms, and PANSS total scores compared with the placebo (*p* < 0.001) [57], and also showed significant improvements in the cognitive domain (*p* < 0.05) [58]. Chaudhry et al. [28] observed a reduction in schizophrenia symptoms on the PANSS total score, but this result was not statistically significant. Furthermore, in secondary analyses of this study, no significant differences were observed in the CGI, GAF, and AIMS [28]. In contrast, ondansetron administration significantly improved visual memory based on visual reproduction, visually paired associations, and the figural memory subtests of the Wechsler Memory Scale-Revised [57]. A study by Kulkarni et al. [58] found no differences in the subscales of the Montgomery–Asberg Depression Rating Scale or PANSS [58]. In patients with treatment-resistant schizophrenia, ondansetron administered for 12 weeks at a dose of 4–8 mg/day, in combination with risperidone [59] or haloperidol [60], showed efficacy, particularly for negative symptoms and cognitive impairment. Patients were assessed using PANSS [59,60], the Wechsler Adult Intelligence Scale (WAIS-R) [59], the Hamilton Rating Scale for Depression (HRSD) [59], as well as CGI-S [60]. Ondansetron, in combination with risperidone, was associated with significant improvements in the PANSS total score and subscales for negative symptoms and cognitive function in comparison with the placebo (*p* < 0.001), and ondansetron administration significantly improved visual memory based on the WAIS subscales (*p* < 0.05) [59]. The combination of ondansetron with haloperidol produced greater improvements in the PANSS scale and subscales for negative symptoms, general psychopathology, and cognitive function at the endpoint compared with the placebo [60]. However, no difference was observed between groups in the subscales for positive symptoms and CGI-S. Furthermore, it has been shown that ondansetron could reduce some side effects induced by antipsychotic treatment, namely parkinsonism, akathisia, behavioral hyperactivity, cardiac, and gastrointestinal side effects [60].

The above studies show that ondansetron may be a potential adjunctive treatment strategy for schizophrenia, particularly for negative symptoms and cognitive impairment. The results of the studies are consistent regarding the duration of observations (12 weeks), the ondansetron doses (4–8 mg/day), and the use of similar scales, including but not limited to the PANSS. The different numbers of patients in the groups do not seem to affect the results obtained.

Zheng (2019) [56] conducted a meta-analysis, in which the efficacy and safety of the strong 5-HT3 receptor antagonist, ondansetron, in schizophrenia was evaluated. Data from five randomized clinical trials, including 184 schizophrenic patients receiving ondansetron (4–8 mg/day) plus neuroleptic treatment and 155 patients receiving a placebo plus neuroleptic treatment, were analyzed. Compared to the placebo, ondansetron was better at reducing the total PANSS score (SMD = −1.06; *p* = 0.04), as well as negative (SMD = −0.96; *p* = 0.01) and general PANSS symptoms (SMD = −0.97; *p* = 0.04), while on the scale of positive symptoms, the change was on the border of statistical significance (*p* = 0.05). Four RCTs investigated the cognitive effects of ondansetron and produced inconsistent results. Ondansetron was better than the placebo at reducing extrapyramidal symptoms and was well-tolerated at the same time [56].

### 4.8. Antihypertensive Drugs

Hypertension is a serious disease, which leads to an increased cardiovascular risk. A cohort study found that the use of dihydropyridine calcium channel blockers reduces the risk of psychiatric hospitalization [91]. The usefulness of certain dihydropyridines (i.e., lercanidipine, amlodipine, and nifedipine) in the treatment of arterial hypertension in patients with psychiatric burdens has been indicated. It has also been shown that taking thiazides does not affect the risk of psychiatric hospitalization. This may suggest that it is worth considering the use of the three above-mentioned dihydropyridines in schizophrenic patients with arterial hypertension.

A randomized, placebo-controlled trial evaluated the effect of telmisartan (80 mg/day) in combination with clozapine or olanzapine over 12 weeks in 43 patients with schizophrenia. There was a significant decrease in the total PANSS score in subjects receiving telmisartan compared to the placebo group (*p* = 0.04) and there were no differences in the range of positive and negative PANSS subscales [61]. It should be noted that patients treated with telmisartan were more likely to smoke and had a lower level of education than the placebo group. The most common adverse reaction from this intervention was dizziness. This may be an indication that the dose of sartan should be reduced in patients without hypertension, while in those with hypertension, the antihypertensive effect is desirable.

It is also worth noting that a decrease in the severity of the psychopathological symptoms of schizophrenia was associated with the group, a significant number of whom were taking clozapine, which proves drug resistance in these patients. The beneficial effect of this intervention may be because angiotensin-II-receptor antagonists can suppress central inflammation by influencing the metabolism of the kynurenine pathway, leading to a reduction in kynurenic acid (KYNA) concentration. This compound is an antagonist of the ionotropic glutamate receptors (GLU) and nicotinic (α-7) receptors. Increased brain levels of KYNA are found in patients with schizophrenia, and its relation to memory impairment and psychotic symptoms has also been demonstrated [92]. Inhibition of KYNA biosynthesis and the decreased kynurenine aminotransferase II (KATII) activity in the central nervous system can be induced with sartans, such as irbesartan, losartan, and telmisartan [93]. This mechanism may be related to the attenuation of the psychopathological symptoms of schizophrenia observed by Fan et al. [61] who added telmisartan to clozapine [61]. This result seems particularly noteworthy with regard to the conclusion of Correll et al.’s [5] systematic review of meta-analytic evidence revealing no significant clinical benefit of the augmentation of clozapine in schizophrenia [5]. However, the aforementioned data may suggest dihydropyridines or sartans as especially valuable options in the treatment of hypertension in schizophrenic patients.

### 4.9. Incretin Drugs

Incretin drugs, which may be helpful both in compensating for metabolic disorders caused by antipsychotic treatment and in the causal treatment of schizophrenia, seem to be a new therapeutic option, offering hope for patients with schizophrenia. They are becoming increasingly used in the treatment of diabetes and metabolic disorders, despite their relatively high cost. However, because of their high effectiveness, they should be of interest to psychiatrists dealing with the treatment of schizophrenia. Schizophrenia is often associated with negative symptoms resulting in decreased motor activity [94]. Additionally, therapy with clozapine or olanzapine lowers the concentration of endogenous glucagon-like peptide 1 (GLP-1), which increases the secretion of glucagon [95]. This, in turn, causes a chronic increase in glucose concentration in the serum of patients, which, together with a low level of physical activity in schizophrenic patients, increases the risk of developing a metabolic syndrome manifested by obesity, insulin resistance, hypercholesterolemia, and arterial hypertension. Clozapine, the last-line drug, induces the above-mentioned complications. It is estimated that currently at least 1/3 of patients do not respond to antipsychotic therapy due to the multiple burdens with many factors of potential drug resistance [94]. In these patients, consideration should be given to initiating clozapine treatment as soon as possible, rather than after other therapeutic options have been exhausted. Potentially, the use of GLP-1 analogs interacting with the incretin system may reduce the risk of clozapine metabolic side effects even before they occur. This would make it possible to use clozapine in more patients in the early stage of the disease.

Mayfield et al. [96] showed in a meta-analysis that liraglutide demonstrates the highest efficacy among incretin drugs in combating obesity [96]. In addition, its use was related to improved cognitive function in studies involving animal models [97]. This may be a promising lead, taking into account the high concentration of GLP-1 receptors in the parietal cortex, hypothalamus, and medulla oblongata [95]. Liraglutide has also been shown to have anti-inflammatory, antioxidant, and anti-apoptotic effects in radiation-induced encephalitis in mice [98]. In turn, the mechanism of its anti-apoptotic effect was correlated with an increase in the expression of transmembrane molecules in the mitochondria, Bcl-2 and Bcl-xl, and a decrease in Bax and Bad [99]. Its use also caused a reduction in the concentration and activity of pro-apoptotic factors, such as NF-ϰB, ICAM-1, caspase-3, and reactive oxygen species (ROS). It is also possible for liraglutide to act as a stimulator of brain stem cells, which seems to be crucial in terms of neuroplasticity processes. This may be important in patients with cerebral ischemia, resulting from comorbid cardiovascular diseases and lifestyle factors. These assumptions may be confirmed by the observation of animals in which the effects of deliberately induced cerebral ischemia were lower in those receiving liraglutide compared to the control group [100]. Liraglutide can, therefore, be considered not only as a drug to limit the side effects of antipsychotics (especially excessive weight gain) but also—in the context of the inflammatory theory of schizophrenia—as an outcome-improving medication. A Larsen et al. [101] study confirmed the metabolic efficacy of liraglutide in improving glucose tolerance, body weight, and cardiometabolic disturbances in patients with schizophrenia spectrum disorders treated with clozapine or olanzapine [101].

## 5. Clinical Significance—Future Directions

Due to the high percentage of schizophrenic patients with drug resistance or an incomplete response to antipsychotic therapy, means to potentiate the efficacy of antipsychotic medications may have significant practical importance, especially in line with the evidence indicating that effectively treated schizophrenic patients comply better when the therapy of coexisting somatic diseases is used [102]. Therefore, expectations of the positive influence of such interventions to increase life expectancy seem to be justified.

Most of these beneficial pharmacological interventions affect the cardiovascular system and metabolic processes. Attention has been paid in recent years to the increased risk of CVD in individuals with schizophrenia. Similar to the general population, CVD is the main reason for mortality, however, the average life expectancy in patients with schizophrenia proves to be 10 years lower [103]. A recently published study showed a 30-year risk of developing CVD in patients with schizophrenia aged 18–59 years without CVD but with two high-risk factors, 20.8% (95% CI: 18.5% ÷ 23.4%) in patients with schizoaffective psychosis, 27.5% (95% CI: 25.3% ÷ 29.8%), and 10.8% in healthy subjects. A high BMI and smoking appeared to be the main predisposing factors. In total, 31.4% were found to be overweight, obesity was present in 45.2% of persons with schizophrenia and in 25.1% and 57.4% of individuals with schizoaffective disorder, respectively, compared to the general population with a prevalence of overweight in 34.5% and obesity in 34.5% [104].

Dysregulation of the HPA axis with hypercortisolemia [103], smoldering inflammation impairing the function of blood vessel endothelium [105], and dysfunction of the autonomic nervous system with a decrease in heart function variability are regarded as the common pathogenetic basis for CVD and schizophrenia [104].

Moreover, body weight increase as a consequence of antipsychotic therapy and stimulation of sympathetic system activity as a consequence of peripheral dopamine receptor blockade may constitute additional somatic risks in schizophrenic patients [104,105]. The list of risk factors for cardiovascular diseases in persons with schizophrenia also includes poverty, poor diet, inactive lifestyle, substandard use of medical care, or nicotine addiction [10]. Augmenting antipsychotic treatment with thoughtfully chosen somatic medications may correct some of these modifiable factors.

It is still unclear whether the described positive effects of add-on therapies apply to the entire group of antipsychotic medications or to only some. Additionally, despite international recommendations suggesting monotherapy as the appropriate form of schizophrenia treatment, concomitant use of several antipsychotics is relatively common practice [106,107,108]. However, neuroleptic polytherapy correlates with a reduction of psychopathology and a decrease in rehospitalization rates, as well as with a significant reduction in mortality rate in patients with schizophrenia [109,110,111]. Nevertheless, this review demonstrates a lack of clinical or cohort studies on the effects of the addition of non-psychotropic drugs to polytherapy with antipsychotics. Therefore, encouraging this type of combination therapy is currently not possible.

Referring the findings of this study to the broader context of drug repositioning in psychiatry, this critical review of the evidence on repurposed drugs as adjunctive treatments for mania (four drugs) and bipolar depression (eight drugs) has revealed a lack of high and moderate quality studies. Therefore, the clinical utility of the results may be not certain, however, adjunctive allopurinol or tamoxifen did improve the efficacy of treatment in mania, and modafinil/armodafinil or pramipexole were effective add-ons in bipolar depression. Interestingly, in the context of our study, improvements associated with celecoxib or N-acetylcysteine were limited only to selected outcomes [112]. What is noteworthy is that only the drug combination paradigm underwent assessment in clinical trials of bipolar disorder.

Among the clinical trials published between 2015 and 2021, which assessed the effects of repurposed medications in patients with major depressive disorder, a significant improvement in depressive symptomatology was associated with armodafinil or esketamine monotherapy as well as with combined therapy with dextromethorphan [14].

## 6. Limitations

There are some limitations in this review. First, data regarding adjunctive therapy in chronic schizophrenia are seldom reported. Currently, available reports mostly focus on finding new molecules which can be used for the treatment of schizophrenia, and which also have a beneficial impact on decreasing general inflammation which accompanies mental diseases. Many studies in which adjunctive therapy has been used have reported the influence of additional drugs on the somatic condition of patients, whereas the influence on the mental state or cognitive functions measured with psychometric scales (e.g., PANSS) has not been fully evaluated.

Another important limitation is the problem with an interpretation of the effectiveness of adjunctive drugs on symptoms of schizophrenia due to variable periods of co-treatment (ranging from 28 days up to 52 weeks). Moreover, there was no follow-up visit in many of the trials, which makes it impossible to judge long-term treatment effectiveness and limits the use of adjunctive therapy in everyday clinical practice. A further limitation is the exclusion of papers published as case reports (only clinical trial results are included) and those published in languages other than English, but in our opinion, making any conclusions on the basis of single cases is pointless. Finally, the number of participants in the clinical trials was enough to formulate some valuable conclusions, but the obtained results need further confirmation in a much bigger population of patients with schizophrenia. Consequently, additional studies with long-term follow-up periods seem to be essential to reinforce the clinical use of adjuvant therapy in schizophrenic patients.

## 7. Summary

Several clinically profitable, non-antipsychotic agents added to the neuroleptic treatment of schizophrenia were reviewed in this paper, among them, aspirin, simvastatin, N-acetylcysteine, memantine (concerning negative symptoms), ondansetron, calcium channel antagonists, dihydropyridine derivatives, and sartans. Liraglutide may also be of interest. Efforts to both potentiate the antipsychotic effect and ameliorate somatic problems accompanying schizophrenia may be regarded as a worthy clinical commitment as well as improving cooperation between psychiatrists and other physicians.

## Figures and Tables

**Table 1:** Study characteristics.

Study	N (Type of the Study)	Intervention/Number of RCT	Results
Non-steroid anti-inflammatory drugs			
Laan [22]	70 (RCT)	Aspirin added to antipsychotic drugs for 3 months (1RCT)	Reduction of the severity of positive and negative symptoms and total PANSS scale 1.57 point; (95% CI 0.06 ÷ 3.07), *p* = 0.018. No influence on the efficiency of cognitive functions.
Other anti-inflammatory drugs			
Sommer [23]	61(RCT)	Simvastatin was added to antipsychotic drugs for 24 months in patients with schizophrenia spectrum disease diagnosed less than 3 years (1RCT)	No significant differences in the severity of psychopathological symptoms after 12 months, however, a significant reduction in the severity after 6 and 24 months (*p* = 0.02 and *p* = 0.04, respectively); no effect on the cognitive functions, performing daily activities of living, and the severity of depression symptoms.
Shen [24] *	339 (M)	RCTs with the use of different statins (6 RCTs)	Significant reduction of positive symptoms of the PANSS scale in the group receiving statins compared to the placebo: SMD = 0.31 (95% CI: 0.01 ÷ 0.62; *p* = 0.05, *n* = 168); reduction of negative symptoms of the PANSS scale: SMD = 0.31 (95% CI: 0.1 ÷ 0.53; *p* = 0.004, *n* = 339). In patients receiving simvastatin, the reduction of the total PANSS score: SMD = 0.42 (95% CI: −0.01 ÷ 0.84; *p* = 0.05, *n* = 89) and the severity of negative symptoms of the PANSS scale: SMD = 0.40 (95 % CI: 0.14 ÷ 0.67; *p* = 0.03, *n* = 220).
Vincenzi [25]	24	Pravastatin added to antipsychotic drugs for 6 to 12 weeks	Significant reduction in positive symptoms of the PANSS scale at 6 weeks from baseline (*p* = 0.01), but it failed to remain after 12 weeks from baseline (*p* = 0.12).
Ghanizadeh [26]	36	Lovastatin added to risperidone or placebo for 8 weeks	Reduction of the PANSS total score after 8 weeks of treatment in patients receiving risperidone and lovastatin, but without statistical significance.
Tajik-Esmaeeli [27]	33	Simvastatin added to risperidone or placebo for 8 weeks	In patients receiving simvastatin, a significant reduction in negative symptom scores from baseline to week 8 (*p* = 0.003) and in total scores (*p* = 0.001). Results were not significant for positive symptoms or general psychopathology scores.
Chaudhry [28]	108	Simvastatin added to antipsychotic medication for 12 weeks	Reduction in total symptoms of schizophrenia, especially in positive and general symptoms, rather than negative symptoms.
Sayyah [29]	40	Atorvastatin added to risperidone or placebo for 6 weeks	No significant changes in schizophrenia symptoms.
Deakin [30]	98	Simvastatin added to antipsychotic medication for up to 6 months	Combination of simvastatin with risperidone caused a significant reduction of the negative syndrome of schizophrenia while such an effect was not observed when simvastatin was added to other antipsychotic drugs.
Antioxidants			
Berk [31]	40 (RCT)	N-Acetylcysteine added to SGA for 24 weeks (1RCT)	Significant reductions of the score on the PANSS positive subscale [md −5.97 (95% CI: −10.44; −1.51), *p* = 0.009], PANSS negative subscale [md −1.83 (95% CI: −3.33; −0.32), *p* = 0.018] and PANSS general subscale [md −2.79 (95% CI: −5.38; −0.20), *p* = 0.035]. Improvement in akathisia intensity observed (*p* = 0.022).
Farokhnia [32]	42 (RCT)	N-Acetylcysteine added to risperidone for 8 weeks (1 RCT)	Improvement on the PANSS general subscale (*p* = 0.006) and on the PANSS general subscale (*p* < 0.001) in the intervention group. No side effects of NACC addition.
Sepehrmanesh [33]	42 (RCT)	N-Acetylcysteine added to first-generation antipsychotic for 12 weeks (1RCT)	Reduction in the positive (F = 5.47, *p* = 0.02) and negative (F = 0.20, df = 1) PANSS subscale scores. No significant difference in the frequency of adverse effects.
PPARγ agonists			
Iranpour [34]	40 (RCT)	Pioglitazone added to risperidone treatment for 8 weeks (1RCT)	Improvement in the severity of positive and negative symptoms (*p* < 0.001).
Neuroprotective drugs			
Çakici [35] *	368 (M)	Estrogens added to antipsychotic drugs for 28 days up to 8 weeks: 4 RCTs in women and 2RCTs in men	Significant reduction of positive and negative symptoms of schizophrenia on the PANSS scale (*p* < 0.0005) compared to the control group of both sexes; general improvement in functioning as measured by the Comprehensive Psychopathological Rating Scale (*p* < 0.0005); effect size value = 0.57.
Kulkarni [36]	36	Estrogens added to antipsychotic treatment for 28 days	Significant improvement on the PANSS positive, negative, and general subscale (*p* < 0.001) was caused by estradiol (100 mdg-patch) in patients receiving antipsychotic medication versus the placebo group. Additionally, estrogen significantly enhanced treatment of acute, severe psychotic symptoms.
Kulkarni [37]	56	Estradiol transdermal added to antipsychotic drugs for 28 days	Transdermal estradiol significantly reduced positive (*p* < 0.005) and general psychopathological symptoms of schizophrenia (*p* < 0.005) compared with women receiving only antipsychotic treatment.
Kulkarni [38]	53	Estradiol 2 mg, orally added to antipsychotic drugs for 14 days	Significant improvement on the PANSS positive, negative, total, and general scales (*p* < 0.0047) in men receiving additionally, orally 2 mg of estradiol in comparison to the placebo group.
Akhondzadeh [39]	32	Estradiol added to haloperidol for 8 weeks	Estradiol added to haloperidol decreased significantly the score of the PANSS positive, negative, and general psychopathological symptoms of schizophrenia (*p* < 0.001) versus the placebo group.
Louzã [40]	44	Conjugated estradiol added to haloperidol for 28 days	Both the conjugated estrogen group and the placebo group showed a similar improvement on the BPRS total score scale (*p* < 0.001).
Ghafari [41]	32	Conjugated estradiol added to antipsychotic treatment for 4 weeks	Significant decrease in positive (*p* = 0.003), negative (*p* < 0.001), general (*p* < 0.001), and total (*p* < 0.001) PANSS scores over 4 weeks in patients receiving a combination of medication versus the control group.
Çakici [35] *	205 (M)	Raloxifene added to antipsychotic drugs for 8 to 24 weeks (5 RTCs)	Improvement in the functioning of patients as measured by the Comprehensive Psychopathological Rating Scale: *p* < 0.002; Reduction of the severity of positive and negative symptoms on the PANSS scale (*p* = 0.027) positively correlated with the time of drug use; effect size value = 0.52.
Kianimehr [42]	23	Raloxifene added to risperidone treatment for 8 weeks	Improvement of positive symptoms on the PANSS scale (*p* < 0.001) and not a significant change of negative and general psychopathology symptoms in women treated with medication versus the placebo.
Khodaie-Ardakani [43]	23	Raloxifene added to risperidone treatment for 8 weeks	The raloxifene group showed significantly greater improvement on the negative subscale (*p* < 0.001), the general psychopathology subscale (*p* = 0.002), and total PANSS score (*p* < 0.001) in comparison to the placebo group.
Kulkarni [44]	26	Raloxifene added to antipsychotic treatment for 12 weeks	Significant reduction in the PANSS general symptom scores for the raloxifene compared with the placebo (β = −3.72; 95%CI, −6.83 to −0.61; *p* = 0.02) groups. Results were not significant for positive and negative symptoms.
Usall [45]	33	Raloxifene added to antipsychotic treatment for 24 weeks	Significant reduction of negative (*p* = 0.027), general symptoms (*p* = 0.003), and total symptomatology (*p* = 0.005) measured with the PANSS in women receiving treatment versus the placebo.
Weiser [46]	100	Raloxifene added to antipsychotic treatment for 16 weeks	No reduction of the severity of positive and negative symptoms and total PANSS scale in severely ill decompensated women treated with a combination of drugs.
NMDA antagonists			
Kishi [47] *	448 (M)	Memantine added to antipsychotic drugs for 6–26 weeks (8 RCTs)	Statistically significant reduction of the severity of negative symptoms on the PANSS scale (*p* = 0.002), especially in younger adult patients, and improvement in the performance of the MMSE test (*p* < 0.0001); insignificant reduction of the severity of positive (*p* = 0.07) and general symptoms on the PANSS scale (*p* = 0.06) and the severity of depression symptoms (*p* = 0.326).
Lucena [48]	10	Memantine added to clozapine for 12 weeks	Significant improvement (*p* < 0.01) in the total BPRS score, its subscales of positive (effect size [ES] = −1.38) and negative (ES = −3.33) symptoms, the CGI score (ES = 1.56), and the MMSE score was observed with memantine as compared with the placebo.
Lieberman [49]	70	Memantine added to atypical antipsychotic drugs for 8 weeks	No significant differences in the severity of schizophrenia symptoms after 8 weeks in patients treated with a combination of drugs versus the placebo. Higher incidence of adverse effects than the placebo.
Lee [50]	26	Memantine added to typical antipsychotic drugs for 12 weeks	Insignificant reduction of the severity of negative symptoms (*p* < 0.12); no improvement of cognitive functions and depressive symptoms.
Rezaei [51]	40	Memantine added to risperidone for 8 weeks	A significantly greater improvement on the negative PANSS subscale, the total score (*p* < 0.001), and the general psychopathology subscale score (*p* = 0.002) than the placebo group; no significant difference in the reduction of positive symptoms score between the two groups (*p* = 0.757).
Omranifard [52]	32	Memantine added to risperidone for 12 weeks	Improvement in the functioning of the intervention group patients was measured using the GAF and QLS scales (*p* < 0.001) versus the placebo.
Fakhri [53]	30	Memantine added to olanzapine for 6 weeks	Significantly improved the positive and negative PANSS score in patients treated with a combination of drugs (*p* < 0.001) versus olanzapine alone; female patients showed significantly better response than males, especially in positive PANSS score. No significant changes in extrapyramidal symptoms.
Veerman [54]	26	Memantine added to clozapine for 26 weeks	Improvement on the PANSS negative subscale score (effect size = 0.29); improved a composite memory score comprising verbal recognition memory and paired associates learning task scores on the CANTAB (effect size = 0.30) in patients with adjunctive therapy versus the placebo.
Mazinani [55]	23	Memantine added to risperidone for 12 weeks	Significant improvement of negative symptoms on the PANSS scale (*p* < 0.001) and cognitive functions; no significant differences between positive and psychopathologic symptoms in the intervention group of patients.
Serotonergic drugs			
Zheng [56] *	184 (M)	Ondansetron added to antipsychotic drugs for 12 weeks (5 RCTs)	Reduction of the severity of negative and general symptoms of schizophrenia (no effect on the severity of positive symptoms): standardized mean difference of the total PANSS score: −0.51.
Akhondzadeh [57]	30	Ondansetron added to risperidone for 12 weeks	The ondansetron group had significantly greater improvement in the negative symptoms, general psychopathological symptoms, and PANSS total scores (*p* < 0.001).
Chaudhry [28]	36	Ondansetron added to antipsychotic treatment for 12 weeks	Reduction of schizophrenia symptoms on PANSS total score versus antipsychotic treatment only, but without statistical significance.
Kulkarni [58]	42	Ondansetron added to atypical antipsychotic drugs for 12 weeks	Ondansetron caused significant improvement both in the cognitive domain (*p* < 0.05) as measured by the Positive and Negative Syndrome Scale and on Total Positive and Negative Syndrome Scale (*p* = 0.06).
Samadi [59]	18	Ondansetron added to risperidone for 12 weeks	Significantly larger improvement in the PANSS overall scale and subscales for negative symptoms and cognition (*p* < 0.001) in patients treated with adjunctive therapy, but without influence on depressive symptoms.
Zhang [60]	58	Ondansetron added to haloperidol for 12 weeks	Significantly greater improvement on PANSS overall scale and subscales for negative symptoms, general psychopathology, and cognition (*p* < 0.05) in patients treated with adjunctive therapy.
Other drugs			
Fan [61]	43 (RCT)	Telmisartan added to clozapine or olanzapine at a dose for 12 weeks (1RCT)	Significant decrease in the PANSS total score compared to the placebo group (*p* = 0.038). No differences between groups in the score on the positive PANSS subscale: *p* = 0.105 (d = 0.39) and on the negative PANSS subscale: *p* = 0.422 (d = 0.18).

abbreviations: M—meta-analysis; RCT—randomized placebo-controlled trial; SGA—second-generation antipsychotic; PANSS—Positive and Negative Syndrome Scale; md—mean difference; CI—confidence interval; PPARγ—peroxisome proliferator-activated receptor gamma; NACC—N-Acetylcysteine; GAF—Global Assessment of Functioning; QLS—Quality of Life Scale; CANTAB—the Cambridge Neuropsychological Test Automated Battery; *—meta analysis.

## Data Availability

Not applicable.

## Supplementary Materials

The following supporting information can be downloaded at: https://www.mdpi.com/article/10.3390/jcm12124012/s1, Supplementary Table S1. Risk of bias-evaluation.

## Abbreviations

Bad—B-cell lymphoma 2-associated death promoter; Bax-B-cell lymphoma 2-associated X Factor; Bcl—B-cell lymphoma 2; Bcl-xl-B-cell lymphoma-extra large; BMI—Body-mass Index; CNS—Central Nervous System; COPD—Chronic Obstructive Pulmonary Disease; COX-1—cyclooxygenase 1; COX-2—cyclooxygenase 2; CRP—C-reactive Protein; CVD—Cardiovascular Diseases; CYP450 2C9—Cytochrome P450 2C9; CYP450 2D6—Cytochrome P450 2D6; ES—Effect Size; E-TR—Early Type Resistance; GLP-1—Glucagon-like peptide 1; HRT—hormone replacement therapy; 5-HT3—5-hydroxytryptamine; ICAM-1—Intercellular Adhesion Molecule 1; IL1-β—Interleukin 1β; IL-6—Interleukin 6; KAT II—Kynurenine aminotransferase II; KYNA—Kynurenic Acid; MMSE—Mini-mental State Examination; NAC—N-acetylcysteine; NAT2—N-acetyltransferase 2; NF-ϰB—Nuclear factor kappa B; NMDA—N-methyl-d-aspartic acid; NSAIDs—Non-steroidal anti-inflammatory drugs; PANSS—Positive and Negative Syndrome Scale; PPARγ—Peroxisome Proliferator-activated receptor γ; RoB—Risk of bias; RCT—Randomized Controlled Trial; ROS—reactive oxygen species; TNF-α—Tumor Necrosis Factor α; UGT1A6—glucuronosyltransferase.
