# Supplementary material for: Augmentation Therapies as Treatments for Coexisting Somatic Problems in Schizophrenia—A Systematic Review
Source: J Clin Med. 2023 Jun 12;12(12):4012. doi: 10.3390/jcm12124012 (PMC10299654; doi:10.3390/jcm12124012)
Supplement: Supplementary file 1 [file jcm-12-04012-s001.zip › jcm-2373522-supplementary.pdf]

Table S1. Risk of bias– evaluation of articles according to method applied by Wiebe A., et al., 2022 [21].

Study	Study popula<br>tion	Drug<br>and dose	Study quality								
Authors	N	mg/d	control	randomiza<br>tion	pre-post<br>comparision	follow-<br>up	registration	experimeter-<br>blinding	analyst-<br>blinding	participant-<br>blinding	overall<br>%
Laan [22]	70	Aspirin<br>1000	+	+	+	+	+	+	+	+	0
Sommer<br>[23]	61	Simvastatin<br>40	+	+	+	-	+	+	+	+	12.5
Shen [24]	339*										
Vincenzi<br>[25]	24	Pravastatin 40	+	+	+	-	+	-	-	-	50
Ghanizadeh<br>[26]	36	Lovastatin 20	+	+	+	-	+	+	+	+	12.5
Tajik-<br>Esmaeeli<br>[27]	33	Simvastatin 40	+	+	+	?	+	+	+	+	12.5
Chaudhry<br>[28]	108	Simvastatin 40	+	+	+	-	+	+	+	-	25
Sayyah<br>[29]	40	Atorvastatin 20	+	+	+	-	-	+	-	-	50
Deakin	98	Simvastatin 40	+	+	+	-	+	-	?	-	50

[30]										
Berk [31]	40	N-acetylcysteine 1g/b.i.d.	+	+	+	+	+	+	+	+	0
Farokhnia [32]	42	N-acetylcysteine 2g	+	+	+	?	+	+	+	+	12.5
Sepehrmanesh [33]	42	N-acetylcysteine 1200 mg	+	+	+	?	+	+	+	+	12.5
Iranpour [34]	40	Pioglitazone 30 mg/d	+	+	+	-	+	+	+	+	12.5
Cakici [35]*	253										
Kulkarni [36]	36	Estrogens 100 mdg (patch)	+	+	+	-	N/A	+	+	+	25
Kulkarni [37]	56	Estradiol 100µg transdermal	+	+	+	-	+	+	+	+	25
Kulkarni [38]	53	Estradiol 2 mg, Orally	+	+	+	-	-	+	+	+	25
Akhondzadeh [39]	32		+	+	+	-	N/A	+	+	+	25

Louzã [40]	44	Estradiol 0.05 mg/d<br>Conjugated estradiol 0.625 mg/d	+	+	+	-	N/A	+	+	+	25
Ghafari [41]	32	Conjugated estradiol 0.625 mg/d	+	+	+	-	-	+	+	+	25
Cakici [35]*	205										
Kianimehr [42]	23	Raloxifene 120 mg/d	+	+	+	-	+	+	+	+	12.5
Khodaie-Ardakani [43]	23	Raloxifene 120 mg/d	+	+	+	-	+	+	+	+	12.5
Kulkarni [44]	26	Raloxifene 120 mg/d	+	+	+	-	+	+	+	+	12.5
Usall [45]	33	Raloxifene 120 mg/d	+	+	+	-	+	+	+	+	12.5
Weiser [46]	100	Raloxifene 120 mg/d	+	+	+	-	+	+	+	+	12.5
Kishi [47] *	257										

Lucena [48]	10	Memantine 20 mg/d	+	+	+	-	+	+	+	+	12.5
Lieberman [49]	70	Memantine 20 mg/d	+	+	+	-	+	+	+	+	12.5
Lee [50]	26	Memantine 20 mg/d	+	+	+	-	+	+	+	+	12.5
Rezaei et [51]	40	Memantine 20 mg/d	+	+	+	-	+	+	+	+	12.5
Omranifard [52]	32	Memantine 20 mg/d	+	+	+	-	+	+	+	+	12.5
Fakhri [53]	30	Memantine 20 mg/d	+	+	+	-	+	+	+	+	12.5
Veerman [54] **	26	Memantine 20 mg/d	+	+	+	-	+	+	+	+	12.5
Mazinani [55]	23	Memantine 20 mg/d	+	+	+	+	+	+	+	+	0
Zheng [56]*	184										
	30	Ondansetron	+	+	+	-	+	+	+	+	12.5

Akhondzadeh [57]		8 mg/d									
Chaudhry [28]	36	Ondansetron 8 mg/d	+	+	+	-	+	+	+	+	12.5
Kulkarni [58]	42	Ondansetron 8 mg/d	+	+	+	-	+	+	+	+	12.5
Samadi [59]	18	Ondansetron 4-8 mg/d	+	+	+	-	+	+	+	+	12.5
Zhang [60]	58	Ondansetron 8 mg/d	+	+	+	-	+	+	+	+	12.5
Fan [61]	61	Telmisartan 80 mg/d	+	+	+	+	+	+	+	+	0

*- meta-analysis, which includes RCT. Each RCT is evaluated separately.
**- crossover study

Based on the Yes/No- criteria, a risk of bias score was evaluated by calculating the percentage of unfulfilled criteria. 0% RoB indicates a high study quality and a 100% RoB a low study quality.
